# Identification of tumor antigens and immune subtypes of glioma for mRNA vaccine development

**DOI:** 10.1002/cam4.4633

**Published:** 2022-03-14

**Authors:** Zhuohui Chen, Xiang Wang, Zhouyi Yan, Mengqi Zhang

**Affiliations:** ^1^ Department of Neurology, Xiangya Hospital Central South University Changsha China; ^2^ National Clinical Research Center for Geriatric Disorders Xiangya Hospital Central South University Changsha China

**Keywords:** glioma, immune antigen, immune landscape, immunotype, mRNA vaccine

## Abstract

Recent evidence suggested that the mRNA vaccine has been effective for many tumors, but its progress in gliomas was slow. In this study, we screened potential tumor antigens and suitable populations for mRNA vaccine to develop mRNA vaccine for glioma. We integrated the normalized RNA sequencing expression data and somatic mutation data from TCGA‐GBM, TCGA‐LGG, and CGGA datasets. Putative antigens in glioma were identified by selecting highly mutated genes with intimate correlation with clinical survival and immune infiltration. An unsupervised partition around medoids algorithm was utilized to stably cluster the patients into five different immune subtypes. Among them, IS1/2 was cold tumor with low tumor mutation burden (TMB), immunogenic cell death (ICDs), and immune checkpoints (ICPs), and IS4/5 was hot tumor with high TMB, ICDs, and ICPs. Monocle3 package was used to evaluate the immune status similarity and evolution in glioma, which identified cluster IS2A/2B within IS2 subtype to be more suitable vaccination receivers. Weighted gene co‐expression network analysis identified five hub immune genes as the biomarkers of patients' immune status in glioma. In conclusion, NAT1, FRRS1, GTF2H2C, BRCA2, GRAP, NR5A2, ABCB4, ZNF90, ERCC6L, and ZNF813 are potential antigens suitable for glioma mRNA vaccine. IS1/2A/2B are suitable for mRNA vaccination.

## Graphical abstract

Novel tumor antigens were identified by choosing overexpressed and mutated genes. Patients with cold tumors which are low immune‐infiltrated were suitable for mRNA vaccine therapy.

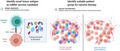

## 1 INTRODUCTION

As one of the most common primary intracranial tumors in adults, glioma is characterized by rapid cell proliferation and angiogenesis and represents 81% of intracranial malignant tumors. The latest statistics show that the prevalence of glioma is 3.2 per 100,000,^1^ and although with a relatively rare prevalence, glioma causes significant mortality and morbidity. According to malignant behaviors, glioma can be divided into WHO grade I‐IV. Glioblastoma is the most malignant tumor, namely grade IV, accounting for about 45% of gliomas, and the 5‐year relative survival rate is about 5%. Glioblastoma has a poor prognosis because its stem cell‐like cells (GSCs) are resistant to conventional treatment.^2^ At present, the effect of targeted therapy for glioma is not significant.^3^ For those glioma patients in low‐level grade, they are sensitive to radiotherapy, chemotherapy, and other treatments, which lead to good prognosis.^4^, ^5^ However, those who are in high grade urgently need a new treatment strategy to improve their survival rate.

As early as the 1990s, Acsadi (1991),^6^ Jiao (1992)^7^discovered and demonstrated the therapeutic potential of mRNA. But because mRNA is unstable and easily degraded by RNA enzymes, it has not attracted much attention. When it came to 1999, the discovery of RNA interference (RNAi) revealed the therapeutic potential of RNA, leading to a boom in RNA vaccine research.^8^ The global spread of the Novel Coronavirus SARS‐COV‐2 in late 2019 has accelerated the development of mRNA vaccine, making it a third‐generation nucleic acid vaccine based on first‐generation attenuated/inactivated vaccines and second‐generation subunit vaccines. The purpose of mRNA vaccines is to transfer RNA into cells for expression and produce protein antigens, thereby inducing an immune response to the antigens to expand the body's immune capacity. mRNA vaccines show high potential in the treatment of tumors. Compared with known tumor vaccines that use peptides, tumor cells, dendritic cells (DCs), and DNA as antigens, mRNA vaccines have many advantages. First, they are easy to design and produce. We can easily modify the mRNA sequence to encode the desired protein and attain faster production.^9^, ^10^, ^11^ Second, mRNA vaccines do not require genetic analysis of cancer, which can reduce much cost.^12^ The third point is safety. mRNA vaccines are not made from pathogen particles or inactivated pathogens, and they do not have the irrelevant sequence rejection and gene integration that often occurs in DNA vaccines. What is more, we can even regulate the half‐life of mRNA through RNA sequence modification or delivery system.^10^, ^11^, ^13^, ^14^ Besides, mRNA vaccines are more immunogenic in human body and can trigger stronger and longer‐lasting immune responses.^15^ The final one is efficacy, current clinical applications have demonstrated the reliability and low side effects of mRNA vaccine. Meanwhile, its simplicity of design makes personalized treatment possible.^10^, ^11^ More importantly, mRNA vaccines have been shown to be effective against other types of cancer, such as melanoma and liver cancer. Preclinical trials have shown that vaccines encoding tumor‐specific antigens can promote antitumor immunity and inhibit the growth of various cancers.^10^, ^16^, ^17^, ^18^, ^19^, ^20^ In conclusion, we believe that mRNA vaccines have great potential in immunotherapy targeting tumor‐specific antigens.

Unfortunately, there are few studies on mRNA vaccines for glioma, and most of them only focused on finding tumor‐specific antigens and immunizing patients against subtypes.^21^, ^22^, ^23^ Based on these findings, we further investigated mRNA vaccines for glioma. By screening potential glioma antigens, we found 10 potential mRNA vaccine antigens. Then we divided glioma patients into five immune subtypes and analyzed the association of immune subtypes with survival, clinical features, and markers of tumor immune microenvironment. Subsequently, we analyzed the molecular characteristics and cell infiltration of the five subtypes. In addition, we also mapped the immune landscape of glioma and identified the co‐expression modules and central genes of glioma immune genes. Our work will pave the way for the development of mRNA vaccine for glioma and the selection of appropriate patients for vaccination.

## 2 RESULTS

### 2.1 Screening of potential tumor antigens of glioma

**FIGURE 1:**
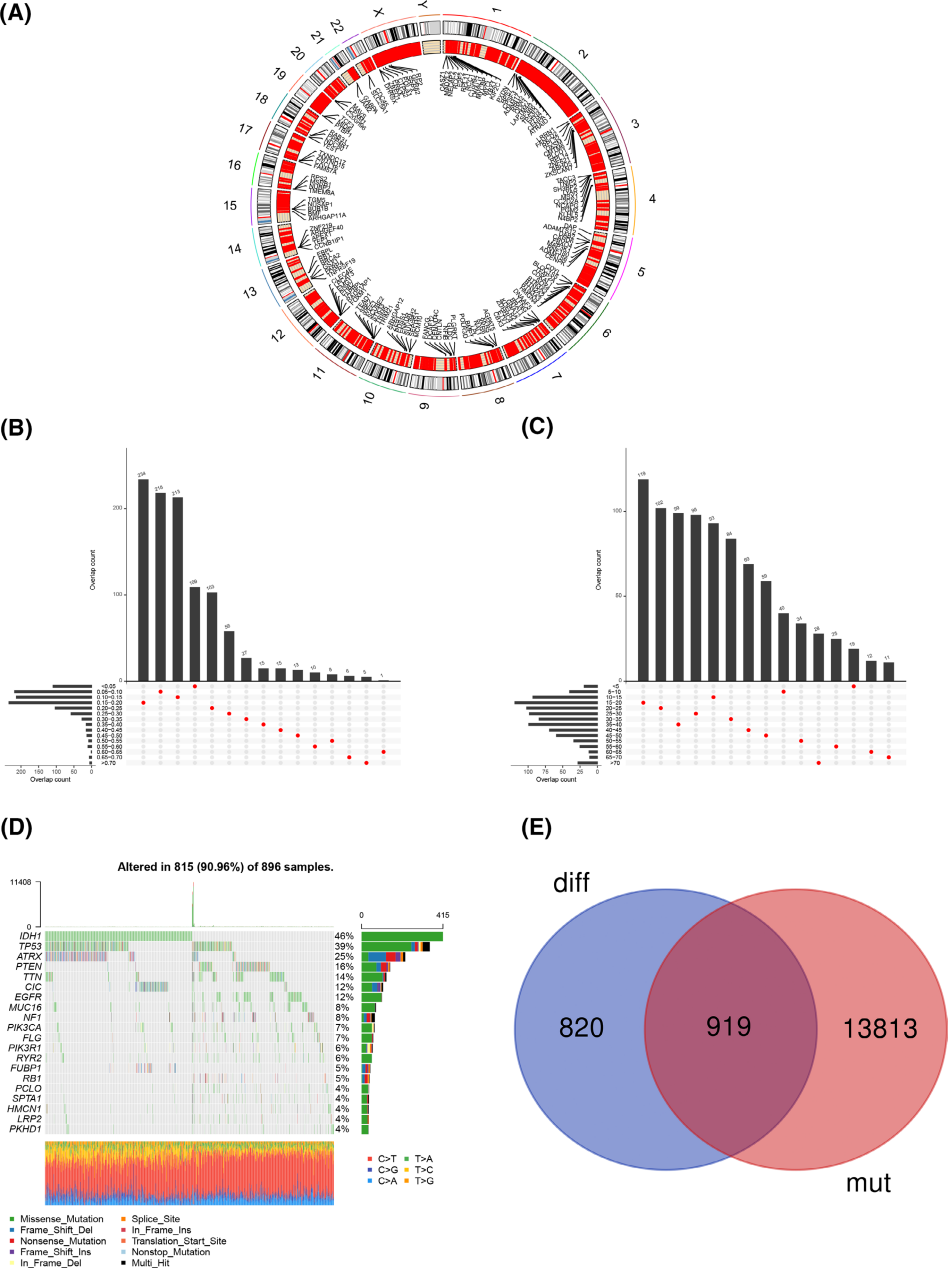
Identification of potential tumor antigens of glioma. (A) Chromosomal distribution of up‐ and downregulated genes in glioma. (B‐E) Identification of potential tumor‐specific antigens in glioma. Overlapping mutated genes distributed in the fraction genome altered group (B) and mutation count group (C) are shown. (D) Genes with the highest mutant frequency are shown. (E) Intersection of upregulated genes and mutant genes

In order to find potential tumor antigens for mRNA vaccine development, differential expression analysis was first performed using the TCGA data. A total of 1739 genes were upregulated in glioma compared with normal tissues. The distribution of upregulated genes in human chromosomes was displayed in Figure 1A. Upregulated genes existed in almost all chromosomes except chromosome Y, and were highly clustered in chromosome 2, 19, and X. Next, mutation analysis was conducted by assessing fraction genome alteration and mutation count and a total of 14,732 mutant genes were identified (Figure 1B and C). Genes with the highest mutation rates, including *IDH1*, *TP53*, *ATRX*, *PTEN*, etc., were displayed in Figure 1D. Notably, gene mutation was quite frequent in glioma and 815 (90.96%) of 896 tumor samples had at least one gene mutation. Finally, 919 potential tumor antigens were identified after the intersection of 1739 upregulated genes and 14,732 mutant genes (Figure 1E).

### 2.2 Identification of tumor antigens associated with glioma prognosis and infiltration of antigen‐presenting cells (APCs)

Survival analysis was then conducted to further screen tumor antigens that might be suitable for mRNA vaccine development. In total, 17 genes were significantly correlated with the overall survival (OS) of glioma patients, among which 14 genes were also significantly associated with the progression‐free survival (PFS) of glioma patients (Figure S1A). Subsequently, associations between gene expression and infiltration of APCs were further analyzed and 10 candidate tumor antigens were finally selected, including NAT1, FRRS1, GTF2H2C, BRCA2, GRAP, NR5A2, ABCB4, ZNF90, ERCC6L, and ZNF813. The distribution of the 10 candidate genes in human chromosomes is displayed in Figure S1B. High expression levels of all these genes were significantly associated with poor OS of glioma patients (Figure S1C‐L). Positive associations between expression of these candidate genes and tumor infiltration of macrophages, dendritic cells, and B cells were observed and NAT1 was best correlated with APCs infiltration (Figure S2A‐J). The associations between expression of candidate genes and APCs infiltration were more significant in lower grade glioma (LGG) compared with that in glioma multiforme (GBM), indicating that mRNA vaccine might exert better treatment effects toward LGG than GBM.

### 2.3 Identification of potential immune subtypes of glioma

**FIGURE 2:**
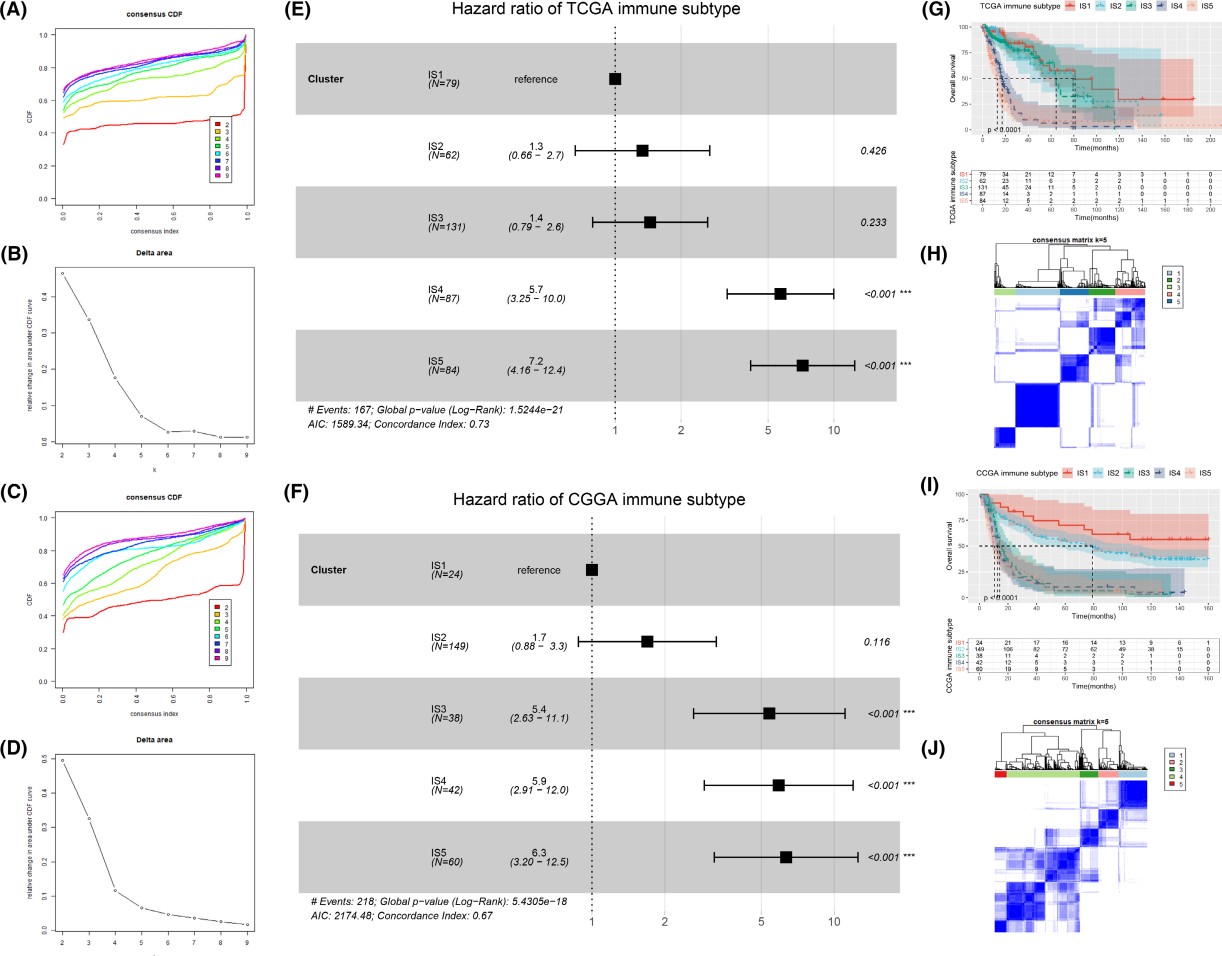
Identification of potential immune subtypes of glioma. (A) Cumulative distribution function curve and (B) delta area of immune‐related genes in the TCGA cohort. (C) Cumulative distribution function curve and (D) delta area of immune‐related genes in the CGGA cohort. Sample clustering heat map in the TCGA cohort (H) and the CGGA cohort (J). (G) Kaplan–Meier curves showing OS of glioma immune subtypes in the TCGA cohort. (E) Hazard ratio of TCGA immune subtype. (I) Kaplan–Meier curves showing OS of glioma immune subtypes in the CGGA cohort. (F) Hazard ratio of CGGA immune subtype

Immunotyping was conducted to identify glioma patients with proper tumor immune microenvironment (TIME) that might benefit more from mRNA vaccine. Expression profiles of 1126 immune‐related genes were extracted from the TCGA and CGGA dataset. By evaluating the consensus matrix and the consensus cumulative distribution function, we chose k = 5 where immune‐related genes seemed to be well clustered (Figure 2A‐D) and obtained five immune subtypes, namely IS1‐IS5 (Figure 2H and J, Table S1, S2). In the TCGA cohort, IS1, IS2, and IS3 showed better prognosis while IS4 and IS5 were associated with worse prognosis (Figure 2E and G). Similarly, in the CCGA cohort, IS3, IS4, and IS5 exhibited worse prognosis while IS1 and IS2 showed better prognosis (Figure 2F and I). Subtype distribution across different grades suggested that patients at different stages were unevenly clustered (Figure S3A‐D). In the TCGA cohort, grade 4 was associated with IS5, while in the CGGA cohort, grade 2 and 3 were associated with IS2. Previous studies have shown that mutations in isocitrate dehydrogenase 1 (IDH1) were significantly associated with longer OS of patients with GBM.^24^ Our results showed that patients with mutant IDH or wild type IDH were also unevenly clustered (Figure S3E‐H). In the TCGA cohort, IDH mutant group was associated with IS1 and IDH wild type group was associated with IS4 and IS5. In the CGGA cohort, IDH mutations tended to be associated with IS2. Taken together, the immune subtypes can predict OS of glioma patients and are correlated with some clinical features as well.

### 2.4 Association of immune subtypes with tumor mutational status

Former studies have established that tumor mutational burden (TMB) is linked with immune status and can predict response to immunotherapies.^25^, ^26^, ^27^ Hence, TMB and number of mutated genes in different immune subtypes were analyzed using the TCGA data. As shown in Figure S4A, TMB in IS4 and IS5 was significantly higher than that in IS1, IS2, and IS3. Consistently, IS4 and IS5 also had a significantly higher number of mutated genes than IS1, IS2, and IS3 (Figure S4B). Ten genes with the highest mutation frequencies in all immune subtypes are shown in Figure S4C. The most frequently mutated gene was *IDH1* (24%), followed by *TP53* (21%) and *ATRX* (14%). These results demonstrated that immune subtypes can predict mutational status in glioma patients, with IS4 and IS5 having higher TMB and mutated genes than IS1, IS2, and IS3, indicating that patients in IS4 and IS5 may be more suitable for anti‐immune checkpoint therapies.

### 2.5 Association of immune subtypes with immune modulators

**FIGURE 3:**
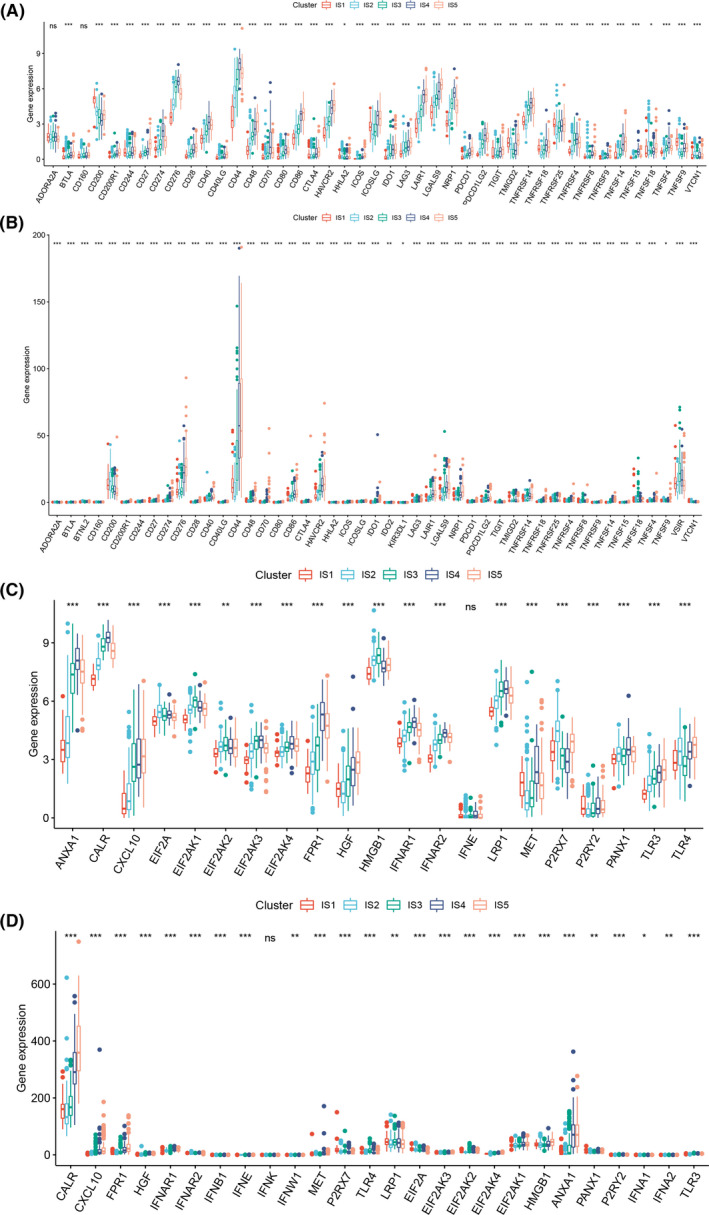
Association of immune subtypes with ICPs and ICD modulators. Differential expression of ICP genes among the glioma immune subtypes in (A) CGGA and (B) TCGA cohorts. Differential expression of ICD modulator genes among the glioma immune subtypes in (C) CGGA and (D) TCGA cohorts

Immune checkpoints (ICPs) and immunogenic cell death (ICD) modulators play a crucial role in anticancer immunity and thus might affect the efficacy of mRNA vaccine. Therefore, we analyzed the expression of ICPs and ICD modulators in different immune subtypes. In the CGGA dataset, a total of 43 ICPs were analyzed and 41 (95.3%) of them were differentially expressed across the immune subtypes (Figure 3A). CD274, CD276, CD44, CD70, NRP1, TNFRSF9, TNFSF15, and TNFSF4 were highly expressed in IS4. In the TCGA dataset, 47 ICPs were analyzed and all of them were differentially expressed across the immune subtypes (Figure 3B). CD244, CD276, CD40, CD44, CD48, CD80, NRP1, and TNFRSF14 were highly expressed in IS4 and IS5. The expressions of ICD modulators was also different across the immune subtypes. In the CGGA dataset, 20 (95.2%) of 21 ICD modulators were distinctly expressed between the immune subtypes (Figure 3C). ANXA1, CALR, FPR1, IFNAR1, IFNAR2, and MET were highly expressed in IS4. In the TCGA dataset, 25 (96.2%) of 26 ICD modulators showed significantly different expression between the immune subtypes (Figure 3D). CALR, FPR1, IFNAR2, EIF2AK1, and ANXA1 were highly expressed in IS4 and IS5. Overall, these results suggest that the immune subtypes were associated with the expression of most ICPs and ICD modulators and that IS1 and IS2 with low expression of ICPs and ICD modulators might show better response to mRNA vaccine.

### 2.6 Cellular and molecular characteristics of the immune subtypes

**FIGURE 4:**
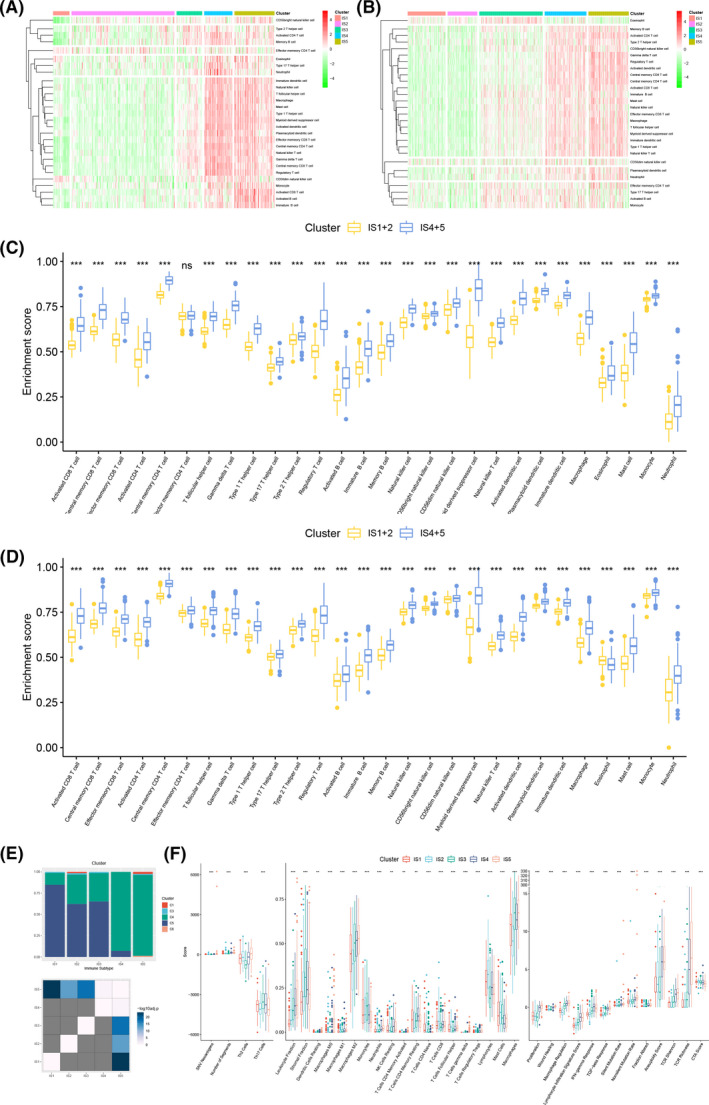
Cellular and molecular characteristics of immune subtypes. Differential enrichment scores of 28 immune cell signatures among glioma immune subtypes in (A) CGGA and (B) TCGA cohorts. Differential immune cell composition in (C) CGGA and (D) TCGA cohorts. (E) The association between the immune subtypes and the six immune categories. (F) Differential enrichment scores of 36 immune signatures with FDR <0.01 among glioma immune subtypes

Since the tumor immune status has a nonnegligible influence on the effectiveness of mRNA vaccine, we used ssGSEA to characterize the immune cell components of the five immune subtypes by scoring 28 previously reported signature genes in both TCGA and CGGA cohorts. In CGGA and TCGA cohorts, we found that IS1 and IS2 showed similar immune cell scores, while IS4 and IS5 showed similar immune cell scores (Figure 4A and B). More interestingly, the immune cell composition was significantly different among IS1, IS2 and IS4, IS5. As shown in Figure 4C, except for effector memory CD4 T cells, the scores of other 27 immune cells including activated CD8 T cells, activated B cells, central memory CD8 T cells were significantly higher in IS4 and IS5 compared to IS1 and IS2 in CGGA cohort. Thus, IS1 and IS2 are immunological “cold” while IS4 and IS5 are immunological “hot” phenotypes. Similar tendency was seen in TCGA cohort (Figure 4D). These results proved that the immune subtype can reflect the immune status of glioma so as to screen suitable patients for mRNA vaccination treatment. Our conjecture that IS1 and IS2 might benefit more from mRNA vaccine treatment is confirmed.

Previously, Thorsson et al. discovered six immune categories (C1‐C6) through the immunogenomic analysis of tumor samples among 33 cancer types, which could define immune response patterns and predict prognosis.^28^ To further confirm the reliability of the immune subtypes in screening patients suitable for mRNA vaccine, we explored the association between the immune subtypes and the six immune categories. As shown in Figure 4E, the distribution of the six immune categories in IS1, IS2, and IS3 was different from that in IS4 and IS5. Specifically, IS1, IS2, and IS3 were mainly composed of C4 (lymphocyte depleted) and C5 (immunologically quiet) while IS4 and IS5 were mainly composed of C4. Given that C4 has worse OS than C5, this result was in accordance with our previous finding that IS4 and IS5 were associated with worse prognosis. C5 was mainly clustered into IS1, IS2, and IS3, confirming that IS1 and IS2 are immunologically cold. Overall, these findings further confirm the efficacy and prognostic value of our immune subtypes. Next, we explored the association between the immune subtypes and 56 previously defined molecular features and identified 36 molecular features with FDR <0.01. As shown in Figure 4F, IS1 and IS2 showed lower scores in most immune cells, except for T follicular helper cell and lymphocytes. IS3 was associated with lower Th2 cells but higher stromal fraction and monocytes. Notably, IS4 and IS5 had higher scores in number of segments, neutrophils, proliferation, wound healing, lymphocyte infiltration, IFN‐γ response, fraction altered, TCR shannon, TCR richness, macrophage M2, and TGF‐β response, indicating an immunologically hot phenotype. In summary, our immune subtypes can reflect immune status in glioma patients and predict response to mRNA vaccine. IS1 and IS2 with an immunologically cold phenotype might be suitable candidates for mRNA vaccine.

### 2.7 The immune landscape of glioma

**FIGURE 5:**
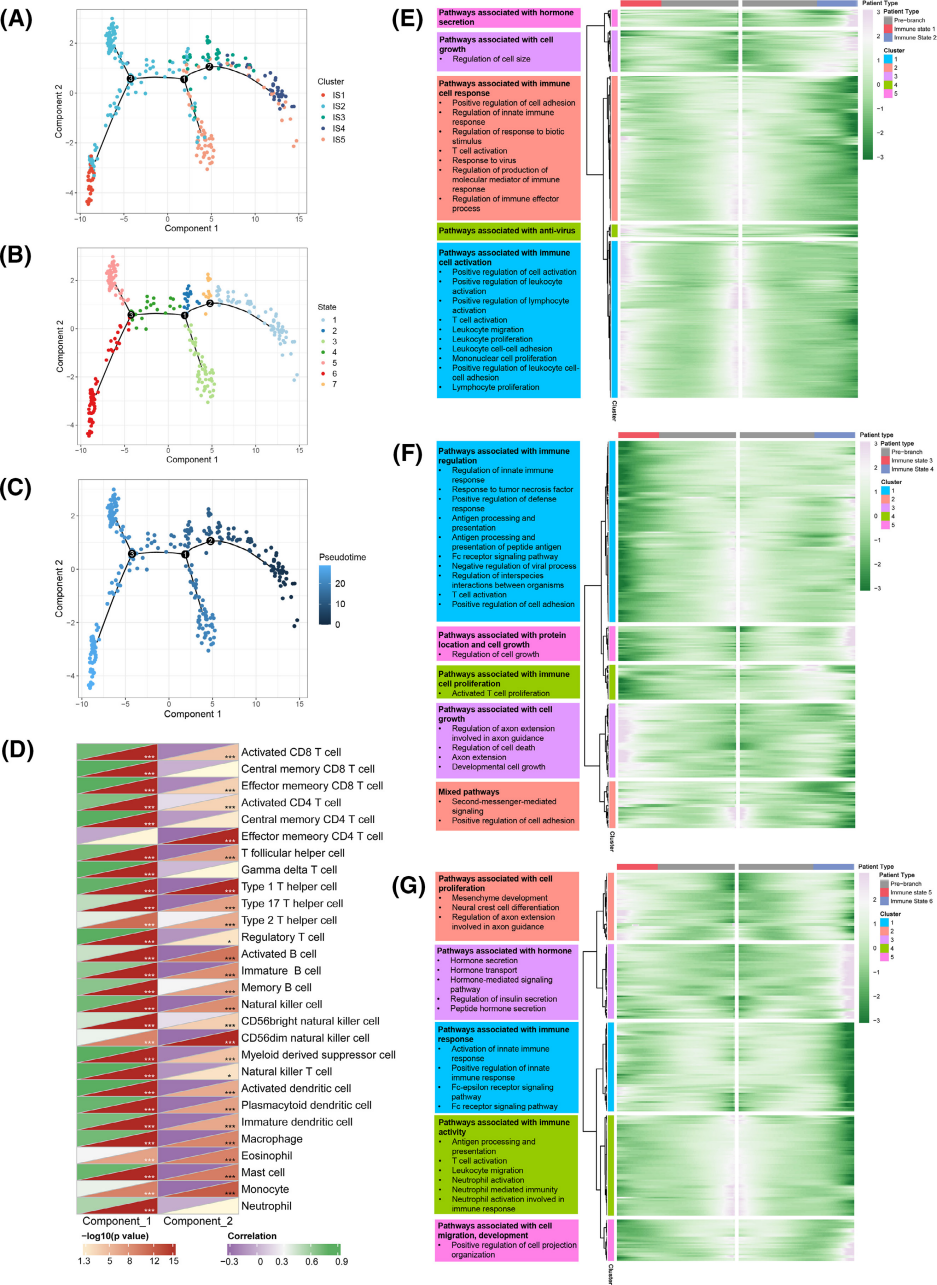
Immune landscape of glioma. (A) Immune landscape of glioma. Each point represents a patient and the immune subtypes are color‐coded. The horizontal axis represents the first principal component and the vertical axis represents the second principal component. (B) Immune landscape of samples from seven different locations depending on branches. (D) Heat map of two principal components with 28 immune cell signatures. (C) Pseudotime analysis of immune landscape of glioma. Analysis of branch point 1 (E), 2 (F), 3 (G)

Immune gene expression profiles extracted from CGGA were used to construct the immune landscape of glioma (Figure 5A and B). As shown in Figure 5D, the horizontal axis was positively correlated with many immune cells, such as activated CD8 T cell, activated CD4 T cell, T follicular helper cell, γδT cell, Th1 cell, regulatory T cell, activated B cell, natural killer cell, activated DC, macrophage, mast cell, etc. On the contrary, the vertical axis was negatively correlated with effector memory CD4 T cell, Th1 cell, and CD56dim natural killer cell. To take a further view on the cellular pathways that might contribute to the differences between the immune states, pseudotime analysis was then conducted where each patient was considered as a single cell (Figure 5C). Subsequently, we conducted GO analysis for the differentially expressed genes of the patients in different directions behind each branch point, three times in total (Table S3–S5). Differentially expressed genes between different immune states at three branch points were clustered into five types according to their expression pattern and subsequent gene enrichment analysis was conducted (Figure 5E‐G). The differentiation direction toward different immune states at branch point 1 might be contributed to pathways associated with hormone secretion, cell growth, immune cell response, anti‐virus, and immune cell activation. Pathways associated with immune regulation, cell growth, and immune cell proliferation might impact the differentiation direction at branch point 2, while pathways associated with cell proliferation, cell migration, hormone, immune response, and immune activity might affect the differentiation direction at branch point 3.

**FIGURE 6:**
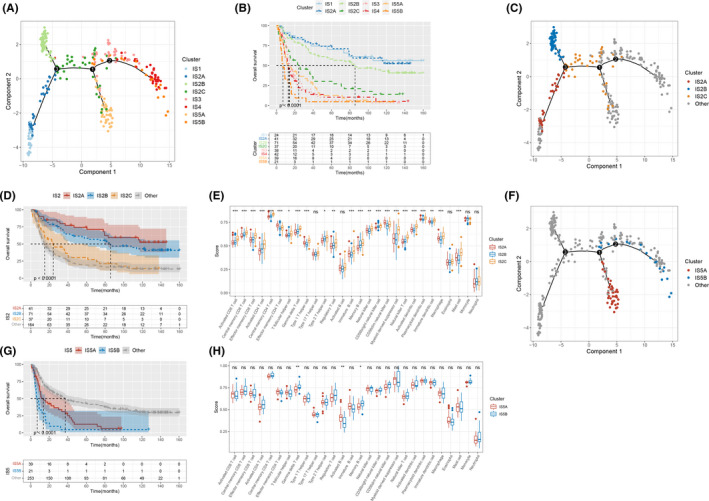
Analysis of the subsets of glioma immune subtypes. (A) Immune landscape of the subsets of glioma immune subtypes. (B) Survival analysis of eight immune subtypes. (C) Immune landscape of the subsets of IS2. (D) Survival analysis of the subsets of IS2 and other. (E) Immune cell infiltration of IS2A, IS2B, and IS2C. (F) Immune landscape of the subsets of IS5. (G) Survival analysis of the subsets of IS5 and other. (H) Immune cell infiltration of IS5A and IS5B

As shown in Figure 5A, heterogeneity was observed not only across different subtypes, but also within the same subtype as well, especially IS2 and IS5. Therefore, IS2 was further classified into IS2A, IS2B, and IS2C and IS5 was classified into IS5A and IS5B based on the distribution location (Figure 6A). Survival analysis revealed that the OS was significantly different among the eight immune subtypes, with IS1 and IS2A showing better prognosis and IS5B showing worse prognosis (Figure 6B). Next, the difference between IS2A, IS2B, and IS2C was explored (Figure 6C–E). The OS of IS2A, IS2B, and IS2C were significantly different, with IS2A and IS2B showing better prognosis compared with IS2C (Figure 6D). There is a growing trend in immune cell infiltration of IS2A, IS2B, and IS2C (Figure 6E). IS2C had higher infiltration of activated CD4 T cell, γδT cell, Th2 cell, memory B cell, natural killer T cell, activated dendritic cell, macrophage, mast cell, regulatory T cell, myeloid‐derived suppressor cell, and so on, suggesting that IS2C was an immunologically hot and immune suppressive phenotype while IS2A and IS2B were immunologically cold. Hence, IS2A and IS2B might be more suitable candidates for the mRNA vaccine than IS2C. The same analysis was conducted in IS5A and IS5B (Figure 6F–H). IS5B had a significantly worse prognosis compared with IS5B (Figure 6G). As for immune cell infiltration, IS5A had higher scores of activated B cell while IS5B had higher scores of γδT cell and memory B cell (Figure 6H). In summary, these results indicate that the immune landscape can identify immune components of glioma patients, predict prognosis and uncover cellular changes behind cancer evolution, which might help in selection of patients that benefit from mRNA vaccine.

### 2.8 Identification immune gene co‐expression modules and immune hub genes of glioma

**FIGURE 7:**
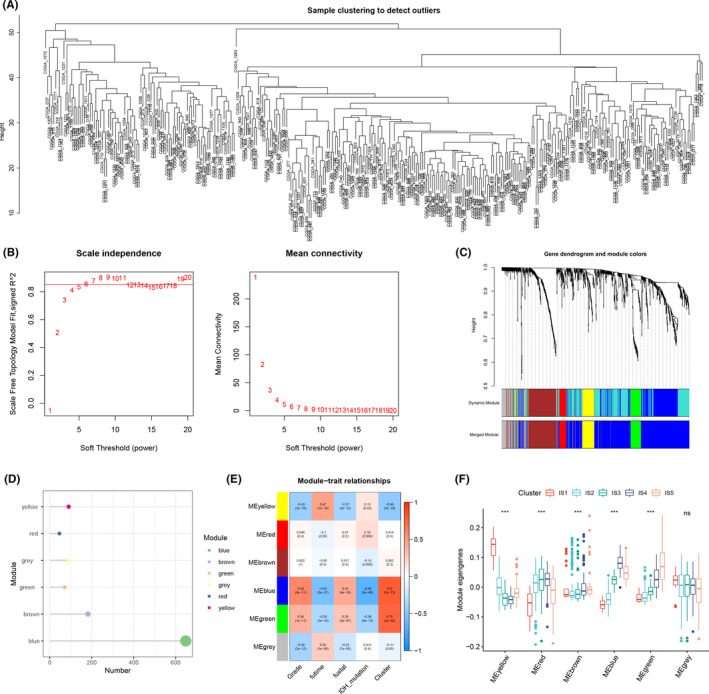
Identification of immune gene co‐expression modules of glioma. (A) Sample clustering. (B) Scale‐free fit index for various soft‐thresholding powers (β) and mean connectivity for various soft‐thresholding powers. (C) Dendrogram of all differentially expressed genes clustered based on a dissimilarity measure (1‐TOM). (D) Gene numbers in each module. (E) Association between the six modules and clinical traits. (F) Differential distribution of feature vectors of each module in glioma subtypes

**FIGURE 8:**
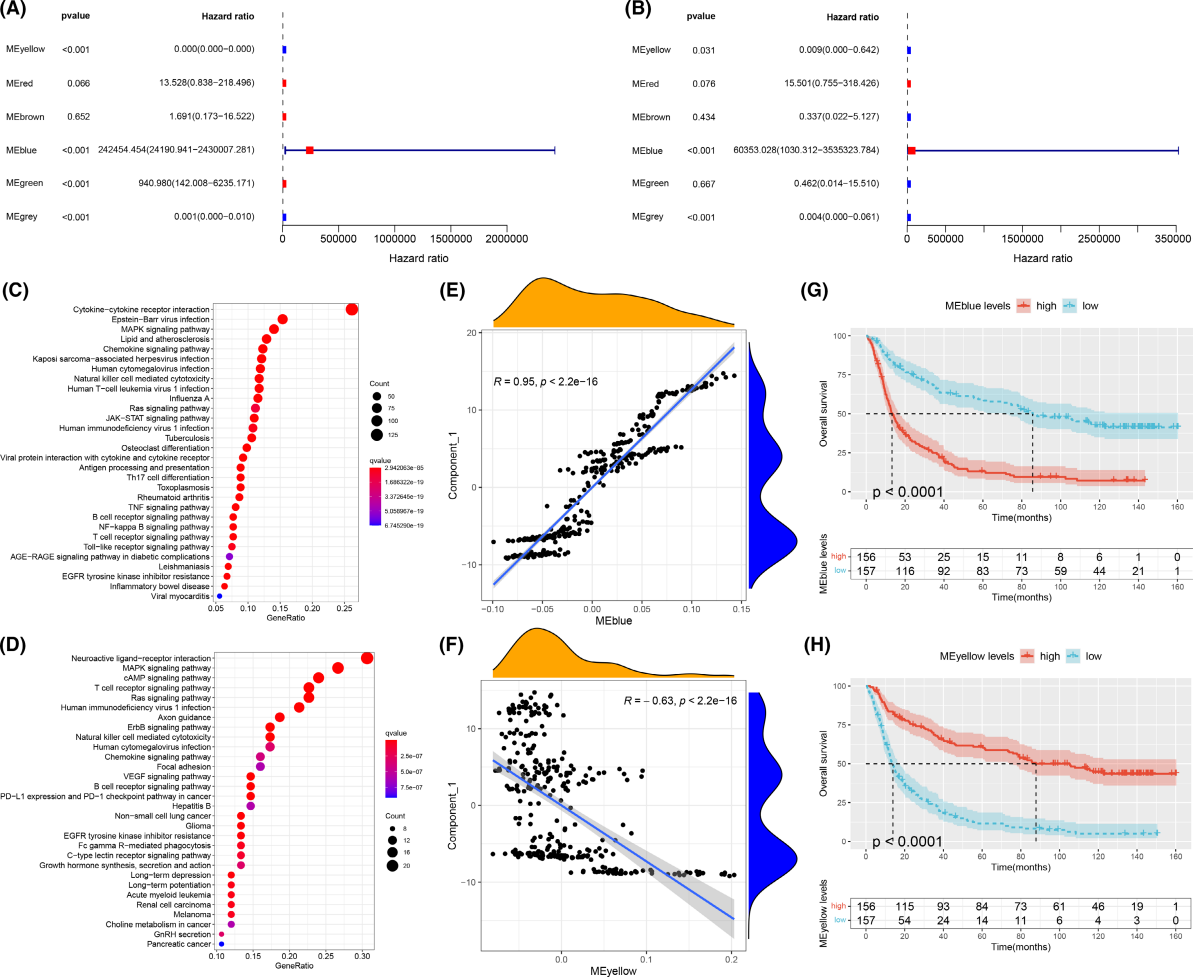
Identification of immune hub genes of glioma. (A) Forest maps of single factor survival analysis of six modules of glioma. (B) Forest maps of multiple factors survival analysis of six modules of glioma. Dot plot showing top 30 KEGG terms in blue (C) module and yellow (D) module. (E) Correlation between blue module feature vector and second principal component in immune landscape. (F) Correlation between yellow module feature vector and second principal component in immune landscape. (G) Differential prognosis in blue module with high and low mean. (H) Differential prognosis in yellow module with high and low mean

Immune gene co‐expression modules were constructed using weighted gene co‐expression network analysis (WGCNA)(Figure 7A) with a soft threshold of 6 in the scale‐free network (Figure 7B). A gene co‐expression network was constructed which was then converted to an adjacency matrix to describe the correlation between different nodes. The adjacency matrix was further converted to a topological overlap matrix (TOM) to quantitatively calculate the similarity in nodes via comparing the weighted correlation among different nodes. Hierarchical clustering with a deep split of four was used to identify modules, in which the minimum module size was set as 30. Based on the hybrid dynamic shear tree, similar modules were merged with abline height as 0.25. The close modules were merged into a new one. The eigengenes of each module were also calculated to identify the expression pattern of each module in different patients (Figure 7C). Therefore, we obtained six gene modules which were respectively represented by yellow, red, brown, blue, green, and gray (Figure 7D). Next, the association between the six modules and clinical traits was explored. As shown in Figure 7E, yellow module and gray module were correlated with lower grade and longer survival, while blue module and green module were associated with higher grade and shorter survival. Besides, the association of the six modules with IDH mutation and the aforementioned immune subtypes was also observed. We further analyzed the distribution of the five immune subtypes in the eigengenes of six modules (Figure 7F). IS1 showed the highest eigengenes in yellow module and the lowest in red module while IS4 and IS5 showed the highest eigengenes in blue and green module. Univariate survival analysis revealed that yellow, blue, green, and gray modules were significantly associated with the survival of glioma patients (Figure 8A). However, in the multivariate survival analysis, only yellow, blue, and gray modules were correlated with survival, among which yellow and gray showed negative correlations with survival and blue showed strong positive correlations with survival (Figure 8B). Since the genes in the gray module did not cluster with the others (Figure 7D), we only focused on blue and yellow modules in the following analysis. Gene enrichment analysis showed that blue module was associated with cytokine–cytokine receptor interaction, Epstein–Barr virus infection, MAPK signaling pathway, etc., while yellow module was associated with neuroactive ligand‐receptor interaction and multiple signaling pathways including MAPK signaling pathway, cAMP signaling pathway, T cell receptor signaling pathway, Ras signaling pathway, etc. (Figure 8C and D). As shown in Figure 8E and F, blue module showed strong positive correlation with component 1 of the immune landscape (*R* > 0.9, *p* < 2.2e‐16) while yellow module was negatively correlated with component 1 (*R* = −0.63, *p* < 2.2e‐16). In the survival analysis, higher expression in the genes of the blue module was significantly correlated with worse prognosis while yellow module showed the opposite (Figure 8G and H). Since blue module showed strong correlation with component 1 and survival, we selected five hub genes (*S100A11*, *TYMP*, *IFI30*, *PLAUR,* and *RAC2*, shown in Table S6) from blue module with module membership (MM) >0.90, which might serve as the marker for selecting patients suitable for mRNA vaccine.

## 3 DISCUSSION

As the most common primary intracranial tumor, glioma is resistant to traditional medical treatment for tumor such as radiotherapy, chemotherapy, and surgical excision because of its immunosuppressive properties.^29^ Current statistics show that the 10‐year survival rate of low‐grade glioma is 47%, with a median survival time of 11.6 years.^30^ For HGG, the median overall survival time for grade 3 glioma patients is approximately 3 years, while the median OS time for grade 4 glioma is only 15 months.^31^ To date, the conventional treatment for gliomas includes surgical resection, temozolomide (TMZ), and radiation, but this is far from enough to combat tumor development.^32^ In recent years, immunotherapy has given new impetus to antitumor therapy for its ability to cross the blood–brain barrier, but more research is needed to prove the feasibility of immunotherapy in treating glioma.^29^ With the success of the mRNA vaccine in clinical trials on other tumors, we see another possibility for glioma treatment. But unfortunately, the application of mRNA vaccine in glioma is still being explored, so we hope our research can further open a breakthrough.

To develop mRNA vaccine for glioma, the first step is to find the appropriate tumor antigen. First of all, we extracted potential tumor antigen gene from TCGA database and got 1739 differentially expressed genes, and then we performed mutation analysis and filtered 14,732 expressed mutant genes. By obtaining the intersection of differentially expressed genes and mutant genes, we got 919 potential tumor antigens. Among these 919 potential tumor antigens, survival analysis was conducted to screen out genes related to the OS and PFS of glioma patients. Finally, 10 candidate antigens that are expected to be used for glioma mRNA vaccine were obtained according to the association with infiltration of APCs, including NAT1, FRRS1, GTF2H2C, BRCA2, GRAP, NR5A2, ABCB4, ZNF90, ERCC6L, and ZNF813. At the same time, infiltration results showed that mRNA vaccine was more suitable for LGG patients than GMB patients. Although further clinical evaluation is needed, some studies have shown the potential of these genes for immunotherapy. Yang et al., for example, found that NR5A2 was highly expressed in glioma tissues and cell lines, and its overexpression was associated with poor prognosis of glioma patients. Further studies showed that NR5A2 could promote cell proliferation and tumor growth via Notch signaling pathway, suggesting that it might be a novel target for immunotherapy in glioma.^33^ Besides, BRCA2 was essential for homologous recombination (HR)‐dependent double‐strand break (DSB) repairing pathway and thus participated in glioma resistance to alkylating agents. Therefore, targeting BRCA2 might be a useful strategy to increase sensitivity to alkylating anticancer drugs.^34^, ^35^ Another study reported that BRCA2 mRNA expression was significantly associated with the overall survival and median survival in patients with LGG.^36^

After screening for potential glioma antigens, we then tried to screen suitable populations for mRNA vaccine. Prior studies have shown that TIME can affect the treatment effect of immunotherapy including mRNA vaccine.^37^, ^38^ Therefore, we then tried to find a reliable method to select patients with proper TIME for mRNA vaccine treatment. Based on the expression profiles of immune‐related genes, glioma patients were classified into five subtypes through consensus clustering. Among the five subtypes, IS4 and IS5 showed worse prognosis compared with IS1, IS2, and IS3, suggesting that immunotyping can act as the predictor for survival of glioma patients. Besides, the immune subtypes were also associated with clinical features, such as tumor grade and IDH mutation. Next, the association between immune subtypes and markers of TIME was investigated. IS4 and IS5 had significantly higher TMB, number of mutated genes and ICD modulators than IS1, IS2, and IS3, suggesting that IS4 and IS5 had higher immune response. ICPs level were significantly higher in IS4 and IS5, indicating that patients in IS4 and IS5 are not suitable for mRNA vaccine alone but might benefit from the combined treatment of immune checkpoint inhibitors and mRNA vaccine. In contrast, IS1 and IS2 with low level of ICPs might show better response to mRNA vaccine. To compare the TIME of different immune subtypes, ssGSEA was used to characterize the immune cell components. With higher scores of most immune cells including activated CD8 T cells, activated B cells, regulatory T cells, etc., IS4 and IS5 were identified as immunologically “hot” and immunosuppressive phenotype while IS1 and IS2 with lower scores of immune cells were identified as immunologically “cold” phenotype. The association between immune subtypes and 56 molecular features also confirmed this finding. Since mRNA vaccine can stimulate immune response in cancer patients, it might be more beneficial to patients with low immune cell infiltration. Hence, IS1 and IS2 were the candidates for mRNA vaccine while IS4 and IS5 were not suitable for mRNA vaccine. To test the reliability of our immune subtypes, we investigated the association between previously reported six immune categories and our immune subtypes and found different distribution of C1‐C6 in different immune subtypes. The proportion of C5 (immunologically quiet) was significantly higher in IS1, IS2, and IS3, confirming that IS1 and IS2 are immunologically cold. In summary, the immune subtypes can not only predict survival, but also help in selecting suitable patients for mRNA vaccine. Specifically, IS1 and IS2 with an immunologically cold phenotype are the suitable candidates for mRNA vaccine.

To obtain an intuitive knowledge of the immune status of glioma patients, immune landscape was constructed using graph learning‐based dimensionality reduction analysis. From the immune landscape, patients can be divided into seven immune states segmented by three branch points. Next, we explored the important cellular pathways that determine immune status shift. The result showed that the differentiation at the three branch points was affected by different pathways. Intervening in these pathways might be a useful strategy for cancer immunotherapy. Overall, patients in the same immune subtype seemed to cluster together while patients in different immune subtypes were distributed in different regions, suggesting the reliability of immune subtypes. However, heterogeneity still existed within the same subtype, especially IS2 and IS5. Therefore IS2 was further classified into IS2A, IS2B, and IS2C, and IS5 was further classified into IS5A and IS5B. Survival analysis showed that IS2A and IS2B showed better prognosis than IS2C, and that IS5B had worse prognosis than IS5A, suggesting that our further classification was reasonable. IS2C showed higher immune cell infiltration than IS2A and IS2B, indicating that IS2C was immune‐hot while IS2A and IS2B were immune‐cold. Thus, IS2A and IS2B might show better response to mRNA vaccine. This result suggests that patients with higher immune cell infiltration tend to have shorter OS, which was in line with above findings. One possible explanation for this might be that patient survival is also influenced by TIME and an immunosuppressive microenvironment contributes to reduced survival time. However, infiltration of most immune cells was similar between IS5A and IS5B. Overall, the immune landscape testified the reliability of the immune subtypes and also allowed us to obtain a more precise classification of glioma patients.

Since immune subtype and immune landscape were not stable across different patient populations, we then conducted further research to find a more stable way that can reflect response to mRNA vaccine. Immune gene co‐expression modules were constructed by WGCNA and six gene modules were obtained, among which yellow, blue, and green modules showed significant correlations with survival. Blue module showed strong positive correlations with component 1 of the immune landscape (*R* > 0.9, *p* < 2.2e‐16) and also significant correlations with survival (*p* < 0.0001). Therefore, five hub genes (*S100A11*, *TYMP*, *IFI30*, *PLAUR,* and *RAC2*) were selected from blue module with MM > 0.90, which might serve as the marker for response to mRNA vaccine.

Several studies also identified potential tumor antigens and immune subtypes in glioma for mRNA vaccine development. Zhong et al. identified four potential tumor antigens (ANXA5, FKBP10, MSN, and PYGL) which were significantly correlated with patient survival and APCs infiltration.^21^ They classified glioma patients into three subtypes, IS1, IS2, and IS3, which were associated with survival, ICPs and ICD modulators but not associated with TMB. Further research showed that IS2 and IS3 were immunologically cold and therefore might benefit from mRNA vaccine. From the immune landscape, IS2 was further classified into IS2A, IS2B, and IS2C, among which IS2A showed lower immune cell infiltration and might show better response to mRNA vaccine. Finally, immune gene co‐expression modules were constructed and hub genes of red and pink modules were selected. Similarly, Ye et al. identified tumor antigens and immune subtypes in LGG for mRNA vaccine development.^22^ However, hub genes of immune gene co‐expression modules were not studied. Another study also used similar methods to identify tumor antigens and immune subtypes in GBM, but they did not construct the immune landscape.^23^ Compared with these studies, we took a further step in the branch point analysis and identified cellular pathways that might impact on the immune status of glioma patients.

In summary, we identified 10 potential tumor antigens associated with survival and APC infiltration, which can be utilized for mRNA vaccine development in glioma. Besides, we classified glioma patients into five immune subtypes which can not only predict prognosis, but also reflect the immune status of glioma patients. IS1 and IS2 with an immunologically cold phenotype might show better response to mRNA vaccine, while IS4 and IS5 with an immunologically hot and immunosuppressive phenotype might not be suitable for mRNA vaccine. In addition, we constructed the immune landscape and identified cellular pathways that affect the immune status of patients. Further classification of immune subtypes was done according to the immune landscape, in which IS2A and IS2B were proved to be more suitable vaccination receiver than IS2C was. Eventually, we built immune gene co‐expression modules and selected five hub genes that might serve as a biomarker indicating response to mRNA vaccine. Overall, our research provided the theoretical basis and would hopefully accelerate the development of mRNA vaccine in glioma.

## 4 MATERIALS AND METHODS

### 4.1 Acquisition of data

The normalized RNA sequencing expression data from 703 samples and 325 samples were collected from TCGA (https://portal.gdc.cancer.gov/) and the CGGA (http://www.cgga.org.cn/) database, respectively. Somatic mutation data annotated by Varscan of TCGA datasets were also downloaded from GDC repository to identify the potential tumor antigens in glioma. A gene list encompassing 1793 immune‐related genes was acquired in ImmPort database (https://www.immport.org/shared/genelists) to identify the immune landscape of glioma. The functions and gene ontology analyses of these immunologically relevant genes were annotated as terms including Antigen Processing and Presentation, Antimicrobials, BCR Signaling Pathway, etc. To undercover the immune features of different immune subtypes, 56 immune traits of glioma samples were exploited from the previously published research. In addition, clinical information regarding the tumor grade, survival information, progression free status, IDH mutation status, etc. of TCGA and CGGA datasets was also extracted to determine the correlation between the clinical parameters and novel antigens and immune subtypes.

### 4.2 Preprocessing of data

Samples from TCGA and CGGA with no clinical information were excluded from the subsequent analyses, with ultimately 444 TCGA samples and 325 CGGA samples included in the following immune subtype identification. Additionally, only 1126 immunologically relevant genes were expressed simultaneously in TCGA and CGGA datasets. To enhance the reliability of the immune subtype classification, only these 1126 genes were utilized to construct different immune subtypes in both datasets. Although not involving all the immunologically relevant genes, the 1126 genes still have covered all the functional and gene ontological annotation terms which guaranteed their representativeness.

### 4.3 Identification of putative antigens

The putative antigens were considered as proteins that were overexpressed and mutated in tumor samples with poor prognosis, and that were highly correlated with antigen‐presenting cells infiltration. In this process, differential expression analyses, univariate Cox regression analyses, and product‐limit method were first utilized. Genes with false discovery rate (FDR) <0.01 and log fold change >1.0 were considered significantly overexpressed; while genes with a hazard ratio (HR) >2, *p* < 0.001 in univariate Cox and log‐rank test *p* < 0.001 were considered clinically relevant. R packages including Limma,^39^ Survminer, Survival were used.

### 4.4 TIMER analyses

As mentioned above, to evaluate the correlation between the putative antigens and antigen‐ presenting cells infiltration status, Tumor Immune Estimation Resource (TIMER, http://timer.cistrome.org/) was utilized in both Lower grade glioma (LGG) and glioma multiforme (GBM) cohort.

### 4.5 Single‐sample GSEA (ssGSEA) analyses

To quantitively predict the immune cells infiltration in the tumor microenvironment, ssGSEA was utilized to calculate the enrichment score of 28 immune cells with different functional statuses (i.e., Activated CD8 T cell, Central memory CD8 T cell, and Effector memeory CD8 T cell) of each patient. The principle of ssGSEA has been described in the previous publication.^40^

### 4.6 Immune subtypes classification

The intersected 1126 immune‐related genes were exploited to cluster the patients and annotate their different immune statuses. Using the partition around medoids algorithm which showed better stability, patients were successfully clustered into five immune statuses (IS) in both TCGA cohort and CGGA validation cohort. Specifically, the distances between patients were calculated using the “1‐Pearson correlation” distance metric. Five‐hundred bootstraps were conducted to enhance the reproducibility of the clustering, with each bootstrap involving 80% of patients in the discovery cohort. We observed the putative clustering number from 2 to 9. The consensus matrix, consensus cumulative distribution function (CDF), and relative change in area under CDF curve were utilized to determine the optimal clustering number.

### 4.7 Construction of immune landscape

Monocle R package was exploited to construct the immune landscape of patients using 700 highly variable immune‐related genes.^41^ Monocle R package was initially designed to construct the evolution landscape of each cell in single cell RNA sequencing. In our present study, we considered individual patients as a single “cell”. Using graph learning‐based dimensionality reduction analysis with a Gaussian distribution in Monocle, the immune status of each “cell” was calculated and visualized. The immune landscape also indicated the difference and correlation among different patients. The maximum number of components were set to four and the dimensional reduction method was set as DDRTress. The immune landscape displayed the patients with different immune subtypes using varied colors. The pseudo‐time analysis was implemented to determine the similarity of immune status among patients. Furthermore, differentially expressed genes were identified by comparing patients extended to different directions from the branch points. KEGG analyses further determined altered pathways contributing to the immune subtype shift among glioma patients.

## CONFLICT OF INTEREST

No potential competing interest was reported by the authors.

## AUTHOR CONTRIBUTIONS

Conceptulization, software, and formal analysis, C.Z.; writing‐orginal draft preparation and visualization, W.X.; data curation, writing‐review and editing, Y.Z.; conceptualization, supervision, funding acquisition, and validation, Z.M.. All authors have read and agreed to the published version of the manuscript.

## Supporting information


Figure S1‐S4

Table S1‐S6

## Data Availability

Public data can be downloaded from following databases including The Cancer Genome Atlas (TCGA, https://portal.gdc.cancer.gov/), Chinese Glioma Genome Atlas (CGGA, http://www.cgga.org.cn/), and Immport database (https://www.immport.org/shared/genelists).
