# Supplementary material for: Identification of tumor antigens and immune subtypes of glioma for mRNA vaccine development
Source: Cancer Med. 2022 Mar 14;11(13):2711–26. doi: 10.1002/cam4.4633 (PMC9249984; doi:10.1002/cam4.4633)
Supplement: Supplementary file 1 — Figure S1‐S4 [file CAM4-11-2711-s001.docx]

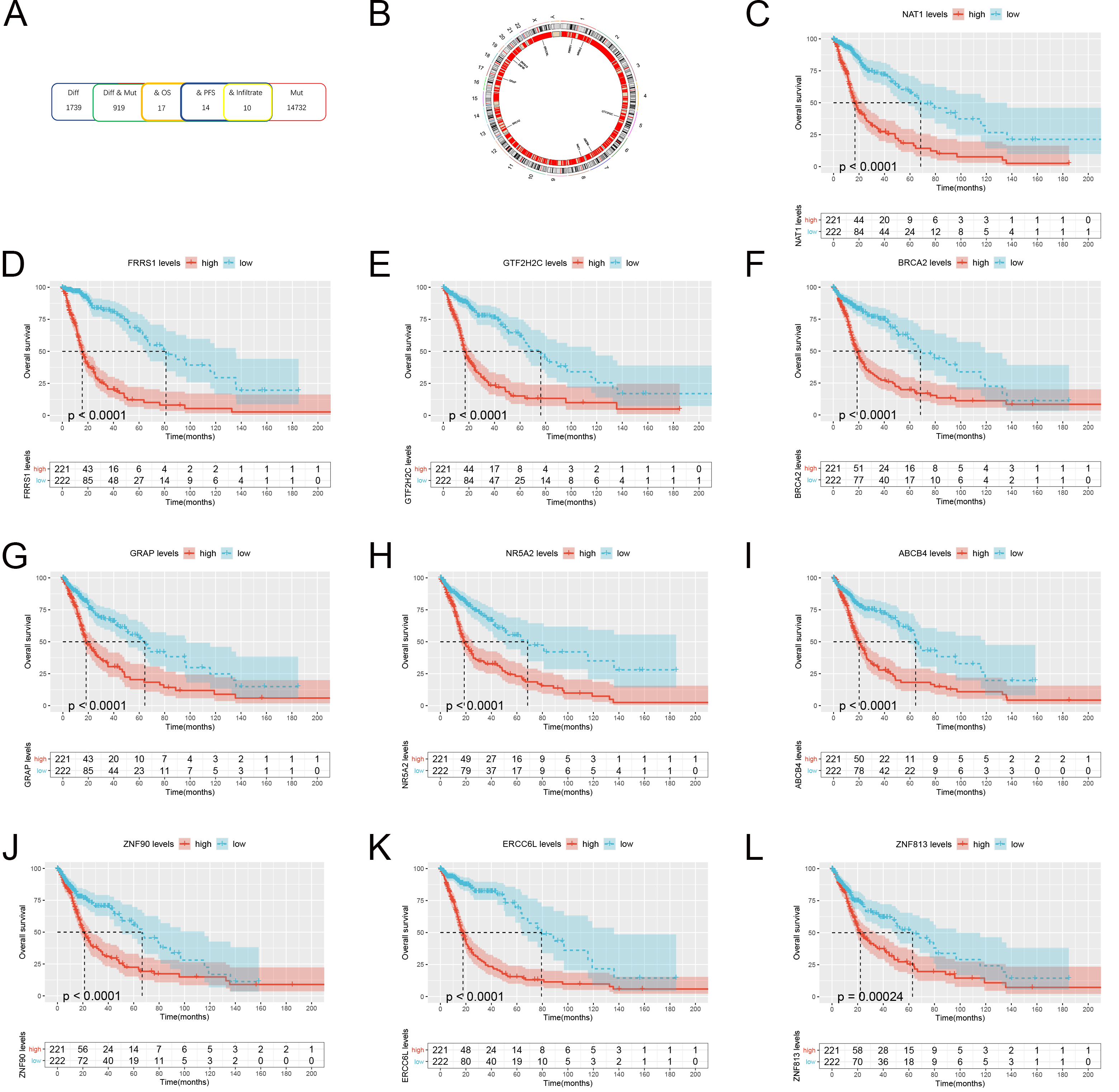


**Figure S1. Identification of tumor antigens associated with glioma prognosis.** (A) Narrow-down analysis of potential tumor antigens with both differentially expressed and mutated features (in a total of 919 candidates), and significant OS and PFS and Infiltration prognosis (in a total of 10 candidates) in glioma. (B) Chromosomal distribution of 10 candidate tumor antigens. (C-L) Kaplan-Meier curves showing OS of glioma patients stratified on the basis of (C) NAT1, (D) FRRS1, (E) GTF2H2C, (F) BRCA2, (G) GRAP, (H) NR5A2, (I) ABCB4, (J) ZNF90, (K) ERCC6L and (L) ZNF813 expression levels.


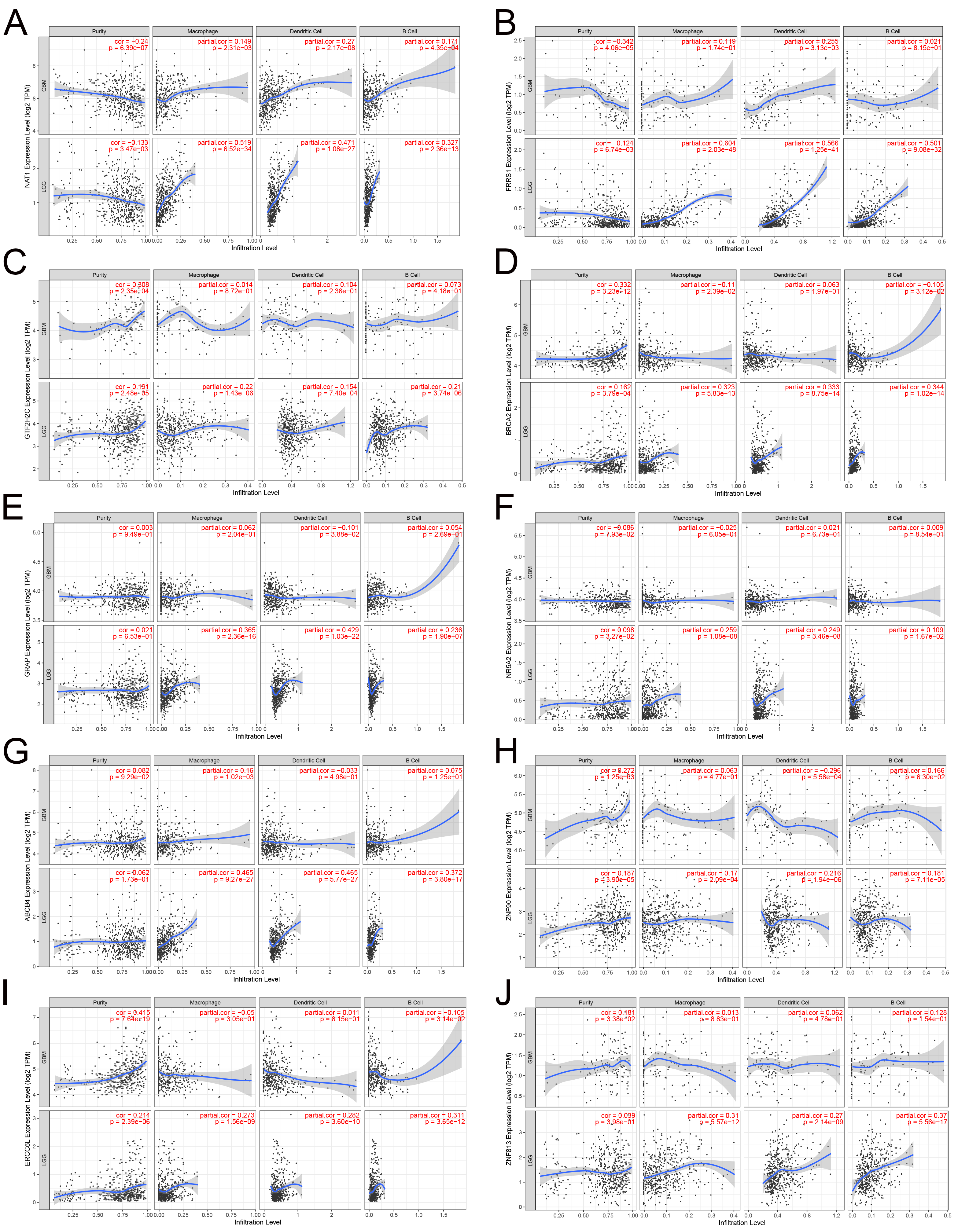


**Figure S2. Identification of tumor antigens associated with antigen-presenting cells.** Association of (A) NAT1, (B) FRRS1, (C) GTF2H2C, (D) BRCA2, (E) GRAP, (F) NR5A2, (G) ABCB4, (H) ZNF90, (I) ERCC6L and (J) ZNF813 expression with the purity of infiltrating cells and amount of macrophages, dendritic cells, and B cells in glioma.


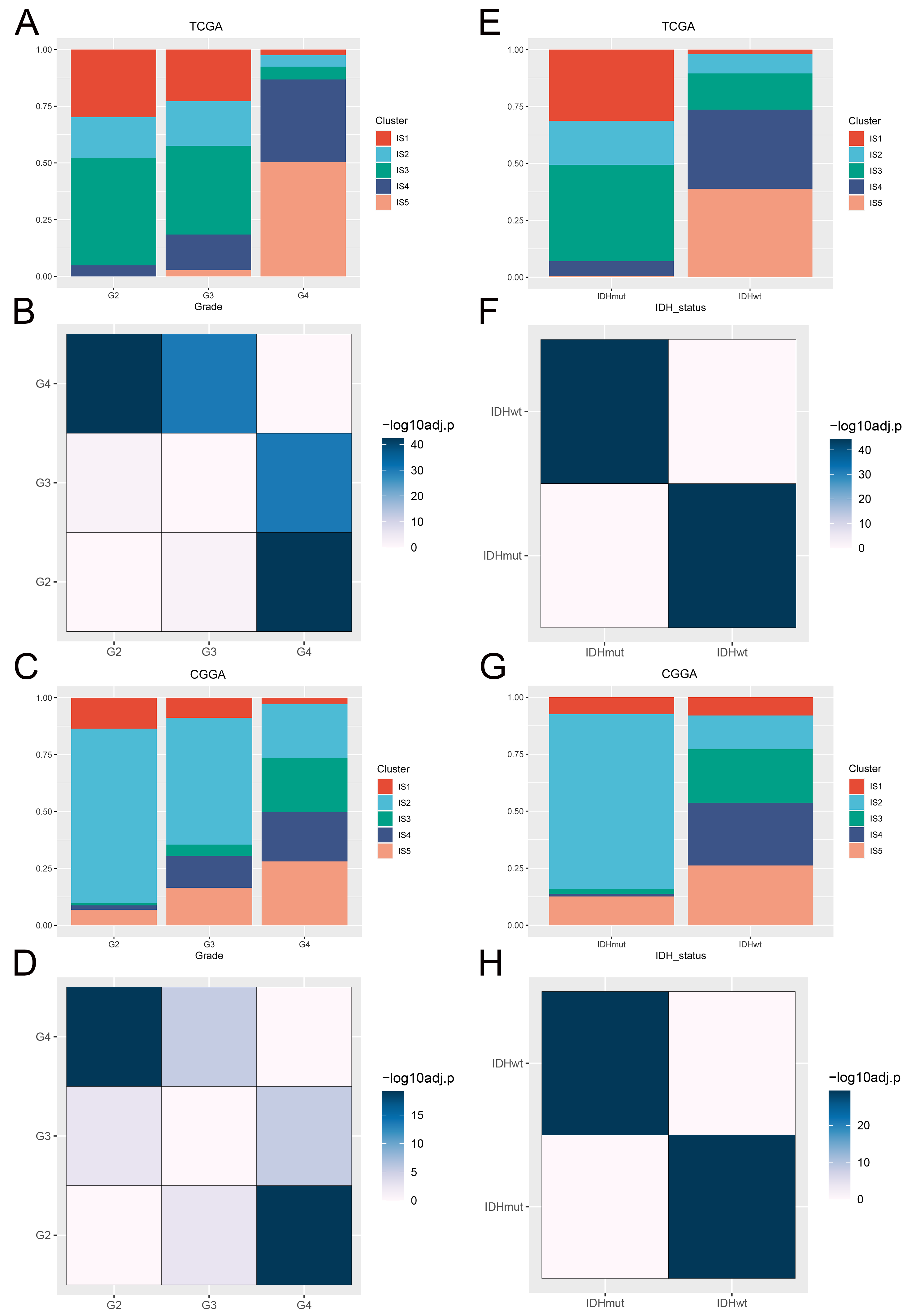


**Figure S3. Identification of potential immune subtypes of glioma.** Distribution of IS1-IS5 across glioma grades in the TCGA cohort (A,B) and the CGGA cohort (C,D). Distribution of IS1-IS5 across glioma IDH_status in the TCGA cohort (E,F) and the CGGA cohort (G,H).


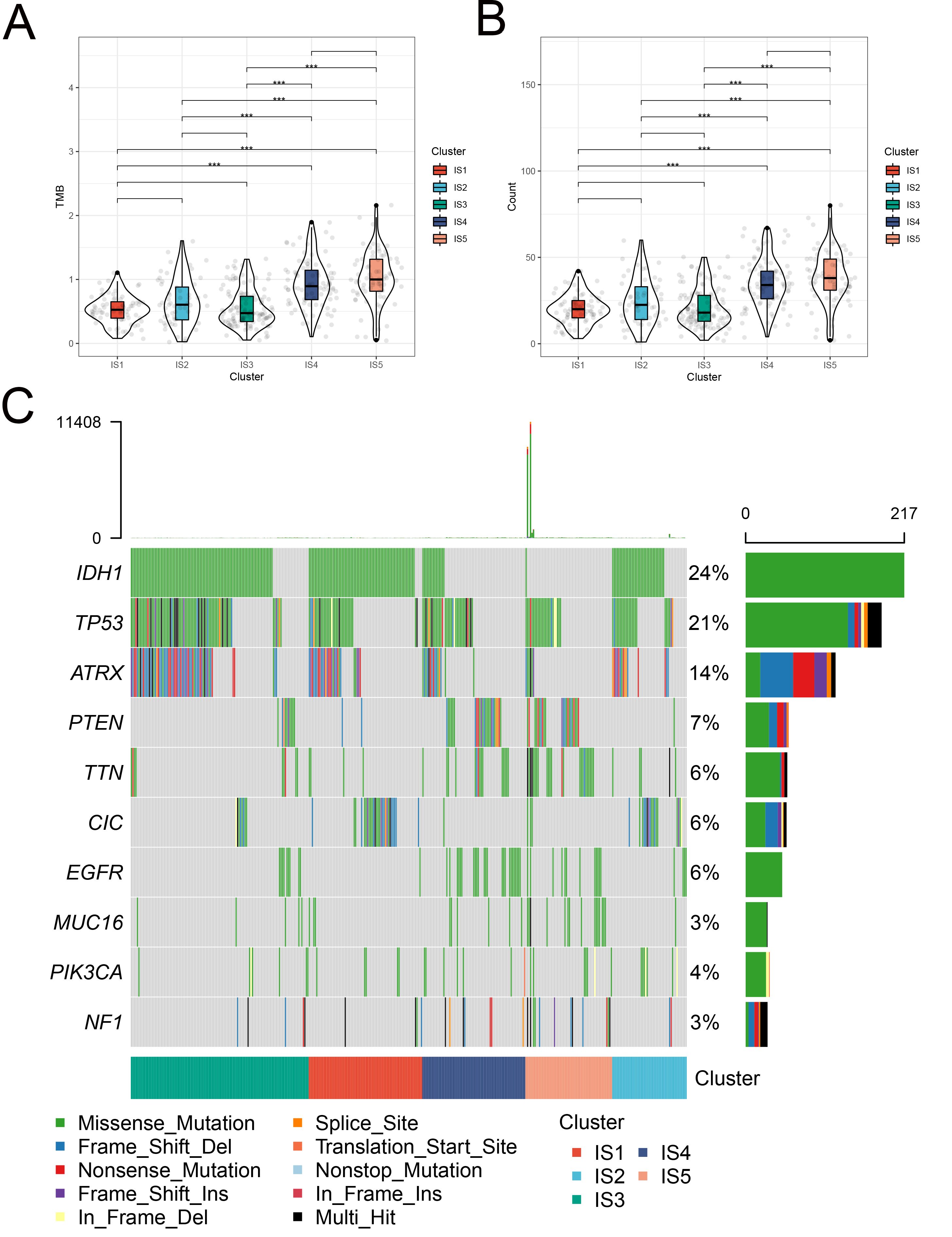


**Figure S4. Association of immune subtypes with TMB and mutation.** TMB (A) and the number of mutated genes (B) in glioma IS1-IS5. (C) Ten highly mutated genes in glioma immune subtypes.
